# Transforming Multidisciplinary Team Meetings (MDTs) in the Mid Mersey Specialist Perinatal Service (MMSPS) to Foster a Psychologically Safe Environment

**DOI:** 10.1192/bjo.2026.11495

**Published:** 2026-06-30

**Authors:** Eleanor Swift, Lyndsey Holt, Megan Woodcock

**Affiliations:** Mersey Care NHS Trust, Liverpool, United Kingdom

## Abstract

**Aims:**

Psychological safety and commitment to excellence are both critical in effective problem solving within healthcare. In the MMSPS MDTs were conducted virtually and there was a lack of consistency in chairing. We noticed tension in discussions and an avoidance of bringing cases to MDT. We explored the reasons for this with the aim of improving the quality of our MDTs leading to better patient care.

**Methods:**

We formed a small working group of the Team Manager, Lead Psychologist and aConsultant Psychiatrist. We conducted a brief ten question survey of all staff in the MMSPS, gathering their views on their experience of and the effectiveness of MDTs. This included yes/no and free text responses. Responses were anonymous and reviewed by the working group only.

**Results:**

We had a high response rate of 69% across a range of professions indicating staff were engaged in this project. 38% of respondents did not feel MDTs were effective in their current format. 54% preferred face to face meetings over virtual. A third did not feel confident speaking during MDTs. Qualitative themes included feelings of conflict, feeling unsupported, contributions not feeling valued, ineffective use of MDT time and unstructured presentations.

We identified that key areas for improvement were psychological safety and MDT structure and implemented multiple strategies to improve these areas. We designed an MDT document including 8 points of “shared understanding” (ground rules) agreed on by the whole team, guidance on which cases to bring to MDT and on the role of the chair. We designed a tool for structuring presentations. We implemented a hybrid model giving the choice of face to face or virtual attendance and agreed the leadership team would chair meetings. We held a launch presentation to introduce the new concept to the team, including sharing a video on psychological safety, and we set a date for re-launch of the MDT.

**Conclusion:**

Feedback from staff members has been overwhelmingly positive. The majority of staff prefer to attend face to face. There has been a notable reduction in conflict within meetings, an improved feeling of safety and improved structure and timekeeping. We are repeating the survey shortly to gather formal feedback on the new process. These strategiescould be replicated by other mental health teams hoping to implement a more psychologically safe approach to MDTs.

